# *cis*-Bis(benzyl­diphenyl­phosphane-κ*P*)dichloridoplatinum(II) dichloro­methane sesquisolvate

**DOI:** 10.1107/S1600536811049269

**Published:** 2011-11-23

**Authors:** Wade L. Davis, Reinout Meijboom

**Affiliations:** a Research Center for Synthesis and Catalysis, Department of Chemistry, University of Johannesburg (APK Campus), PO Box 524, Auckland Park, Johannesburg, 2006, South Africa

## Abstract

The asymmetric unit of the title compound, [PtCl_2_(C_19_H_17_P)_2_]_2_·3CH_2_Cl_2_, contains two complex mol­ecules and three dichloro­methane solvent mol­ecules, two of which are disordered over various positions. The Pt^II^ complexes reveal a slightly distorted square-planar geometry with average Pt—P and Pt—Cl bond lengthss of 2.252 (8) and 2.363 (8) Å, respectively, and average P—Pt—P and Cl—Pt—Cl angles of 99.17 (8) and 87.1 (7)°, respectively.

## Related literature

For a review of related compounds, see: Spessard & Miessler (1996 ▶). For related compounds, see: Johansson *et al.* (2002) ▶. For the synthesis of the starting materials, see: Drew & Doyle (1990 ▶).
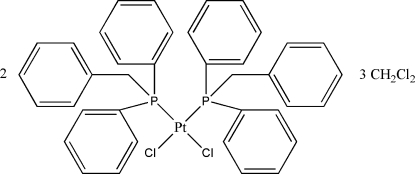


## Experimental

### 

#### Crystal data

[PtCl_2_(C_19_H_17_P)_2_]_2_·3CH_2_Cl_2_
*M* *_r_* = 1891.92
Triclinic, 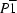

*a* = 11.4087 (9) Å
*b* = 18.6187 (14) Å
*c* = 19.3802 (15) Å
α = 108.079 (2)°
β = 100.438 (2)°
γ = 99.438 (2)°
*V* = 3740.4 (5) Å^3^
*Z* = 2
Mo *K*α radiation
μ = 4.22 mm^−1^
*T* = 100 K
0.27 × 0.22 × 0.16 mm

#### Data collection

Bruker APEX DUO 4K CCD diffractometer
Absorption correction: multi-scan (*SADABS*; Bruker, 2008 ▶) *T*_min_ = 0.395, *T*_max_ = 0.551
101998 measured reflections
18701 independent reflections
17589 reflections with *I* > 2σ(*I*)
*R*_int_ = 0.029

#### Refinement

*R*[*F* ^2^ > 2σ(*F* ^2^)] = 0.020
*wR*(*F* ^2^) = 0.049
*S* = 0.95
18701 reflections
866 parameters
1 restraint
H-atom parameters constrained
Δρ_max_ = 1.60 e Å^−3^
Δρ_min_ = −1.55 e Å^−3^

### 

Data collection: *APEX2* (Bruker, 2010 ▶); cell refinement: *SAINT* (Bruker, 2008 ▶); data reduction: *SAINT*; program(s) used to solve structure: *SIR97* (Altomare *et al.*, 1999 ▶); program(s) used to refine structure: *SHELXL97* (Sheldrick, 2008 ▶); molecular graphics: *DIAMOND* (Brandenburg & Putz, 2005) ▶; software used to prepare material for publication: *publCIF* (Westrip, 2010 ▶) and *WinGX* (Farrugia, 1999 ▶).

## Supplementary Material

Crystal structure: contains datablock(s) global, I. DOI: 10.1107/S1600536811049269/kp2365sup1.cif

Structure factors: contains datablock(s) I. DOI: 10.1107/S1600536811049269/kp2365Isup2.hkl

Additional supplementary materials:  crystallographic information; 3D view; checkCIF report

## Figures and Tables

**Table 1:** Selected geometric parameters (Å, °)

Pt1—P2	2.2436 (6)
Pt1—P1	2.2630 (6)
Pt1—Cl1	2.3602 (6)
Pt1—Cl2	2.3663 (5)
Pt2—P3	2.2505 (5)
Pt2—P4	2.2505 (5)
Pt2—Cl3	2.3531 (5)
Pt2—Cl4	2.3713 (5)

**Fig. 1.**
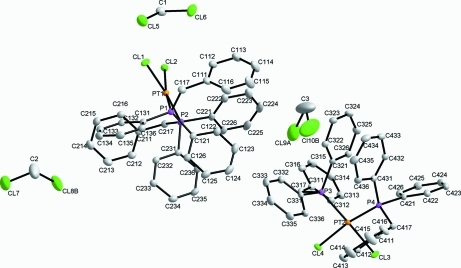
The structure of the title compound showing 50% probability displacement ellipsoids. For the C atoms, the first digit indicates ring number and the second digit indicates the position of the atom in the ring. H atoms have been omitted for clarity. Single orientations (more populated one) are plotted for the disordered solvent molecules.

**Fig. 2.**
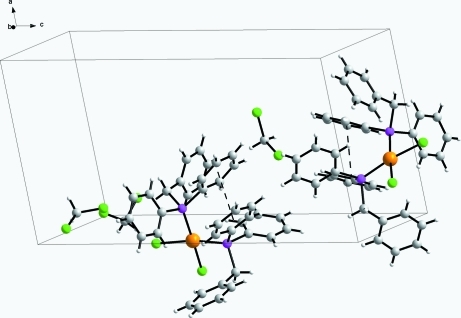
The intramolecular π-stacking between phenyl rings of the title compound. The interaction is indicated by a dashed line.

## supplementary crystallographic information

### Comment

Transition metal complexes containing phosphane, arsine and stibine ligands are widely being investigated in various fields of organometallic chemistry (Spessard & Miessler, 1996). As part of a systematic investigation involving complexes with the general formula *cis/trans*-[*MX*_2_(*L*)_2_] (*M* = Pt or Pd; *X* = halogen, Me, Ph; *L* = group 15 donor ligand), crystals of the title compound, were obtained.

[PtCl_2_(*L*)_2_] (*L* = tertiary phosphane, arsine or stibine) complexes can conveniently be prepared by the substitution of 1,5-cyclooctadiene (COD) from [PtCl_2_(COD)]. The title compound, *cis*-[PtCl_2_(PBzPh_2_)_2_], revals distorted square-planar coordination (Fig. 1 and Table 1) and the Pt atom is slightly elevated out of the coordinating atom plane. All bond angles in the coordination environment of the metal centre deviate significantly from what would be expected for a square-planar geometry. The wide P1—Pt1—P2 angle of 99.2 (2)° and the narrow Cl1—Pt1—Cl2 angle of 87.6 (2)° are a reflection of the steric impact of the two bulky phosphane ligands being in close proximity.

The title compound compares well with other closely related Pt^II^ complexes from the literature containing two chloro and two tertiary phosphane ligands in a *cis* geometry. The average Pt—Cl and Pt—P bond distances of 2.2519 (6) and 2.3627 (6) Å, respectively, fit well into the typical range for complexes of this kind. The title compound crystallises as a solvated complex which is common for these type of Pt^II^ complexes (Johansson *et al.*, 2002). In addition, intramolecular π-stacking between phenyl rings, with distances of 3.5252 (3) and 3.5333 (2) Å, are observed (Fig. 2).

### Experimental

Dichloro(1,5-cyclooctadiene)platinum(II), [PtCl_2_(COD)], was prepared according to the literature procedure of Drew & Doyle (1990). A solution of benzyldiphenylphosphane (55.3 mg, 0.2 mmol) in dichloromethane (2 mL) was added to a solution of [PtCl_2_(COD)] (28.6 mg, 0.1 mmol) in dichloromethane (3 mL). The initial colourless solution turned light-yellow and immediately colourless. Slow evaporation of the solvent gave colourless crystals of the title compound.

### Refinement

The aromatic and methylene H atoms were placed in geometrically idealized positions (C—H = 0.95–0.98) and constrained to ride on their parent atoms with *U*_iso_(H) = 1.2*U*_eq_(C). Large thermal motion of the dichloromethane solvate molecules, held only by weak intermolecular hydrogen bonding, is observed. This was treated isotropically as distorted over 2 partially occupied sites.

### Crystal data

**Table:**

[PtCl_2_(C_19_H_17_P)_2_]_2_·3CH_2_Cl_2_	*Z* = 2
*M**_r_* = 1891.92	*F*(000) = 1867.5
Triclinic, *P*1	*D*_x_ = 1.68 Mg m^−^^3^
Hall symbol: -P 1	Mo *K*α radiation, λ = 0.71073 Å
*a* = 11.4087 (9) Å	Cell parameters from 9569 reflections
*b* = 18.6187 (14) Å	θ = 2.3–28.4°
*c* = 19.3802 (15) Å	µ = 4.22 mm^−^^1^
α = 108.079 (2)°	*T* = 100 K
β = 100.438 (2)°	Cuboid, colourless
γ = 99.438 (2)°	0.27 × 0.22 × 0.16 mm
*V* = 3740.4 (5) Å^3^	

### Data collection

**Table:**

Bruker APEX DUO 4K CCD diffractometer	18701 independent reflections
Radiation source: sealed tube	17589 reflections with *I* > 2σ(*I*)
graphite	*R*_int_ = 0.029
Detector resolution: 8.4 pixels mm^-1^	θ_max_ = 28.4°, θ_min_ = 1.1°
φ and ω scans	*h* = −15→15
Absorption correction: multi-scan (*SADABS*; Bruker, 2008)	*k* = −24→24
*T*_min_ = 0.395, *T*_max_ = 0.551	*l* = −25→25
101998 measured reflections	

### Refinement

**Table:**

Refinement on *F*^2^	Primary atom site location: structure-invariant direct methods
Least-squares matrix: full	Secondary atom site location: difference Fourier map
*R*[*F*^2^ > 2σ(*F*^2^)] = 0.020	Hydrogen site location: inferred from neighbouring sites
*wR*(*F*^2^) = 0.049	H-atom parameters constrained
*S* = 0.95	*w* = 1/[σ^2^(*F*_o_^2^) + (0.0192*P*)^2^ + 8.210*P*] where *P* = (*F*_o_^2^ + 2*F*_c_^2^)/3
18701 reflections	(Δ/σ)_max_ = 0.011
866 parameters	Δρ_max_ = 1.60 e Å^−^^3^
1 restraint	Δρ_min_ = −1.55 e Å^−^^3^

### Special details

**Table:**

Experimental. The intensity data was collected on a Bruker Apex DUO 4 K CCD diffractometer using an exposure time of 20 s/frame. A total of 3086 frames were collected with a frame width of 0.5° covering up to θ = 28.42° with 99.3% completeness accomplished.
Geometry. All e.s.d.'s (except the e.s.d. in the dihedral angle between two l.s. planes) are estimated using the full covariance matrix. The cell e.s.d.'s are taken into account individually in the estimation of e.s.d.'s in distances, angles and torsion angles; correlations between e.s.d.'s in cell parameters are only used when they are defined by crystal symmetry. An approximate (isotropic) treatment of cell e.s.d.'s is used for estimating e.s.d.'s involving l.s. planes.
Refinement. Refinement of *F*^2^ against ALL reflections. The weighted *R*-factor *wR* and goodness of fit *S* are based on *F*^2^, conventional *R*-factors *R* are based on *F*, with *F* set to zero for negative *F*^2^. The threshold expression of *F*^2^ > σ(*F*^2^) is used only for calculating *R*-factors(gt) *etc*. and is not relevant to the choice of reflections for refinement. *R*-factors based on *F*^2^ are statistically about twice as large as those based on *F*, and *R*- factors based on ALL data will be even larger.Highly disordered solvate molecules were observed, resulting in residual electron density around the Cl atoms. Different disordered models, however, resulted in unstable refinement cycles.

### Fractional atomic coordinates and isotropic or equivalent isotropic displacement parameters (Å^2^)

**Table:**

	*x*	*y*	*z*	*U*_iso_*/*U*_eq_	Occ. (<1)
Cl5	0.09600 (7)	0.53139 (4)	0.11524 (5)	0.04068 (16)	
Cl6	0.16152 (6)	0.44051 (5)	0.20840 (4)	0.03724 (15)	
C1	0.0417 (2)	0.47117 (15)	0.16229 (16)	0.0291 (5)	
H1A	−0.0202	0.4250	0.1258	0.035*	
H1B	0.0012	0.4997	0.1995	0.035*	
Pt2	0.426409 (6)	0.842345 (4)	1.116009 (4)	0.01044 (2)	
Cl3	0.52446 (5)	0.90607 (3)	1.24390 (3)	0.02036 (10)	
Cl4	0.31192 (5)	0.93912 (3)	1.13021 (3)	0.01753 (9)	
P3	0.30995 (5)	0.78101 (3)	0.99824 (3)	0.01162 (9)	
P4	0.56353 (5)	0.76804 (3)	1.11485 (3)	0.01164 (9)	
C311	0.07713 (19)	0.74922 (12)	1.03150 (12)	0.0163 (4)	
C312	0.1177 (2)	0.76454 (12)	1.10846 (12)	0.0172 (4)	
H31	0.1929	0.8011	1.1360	0.021*	
C313	0.0491 (2)	0.72681 (13)	1.14505 (13)	0.0205 (4)	
H31A	0.0782	0.7371	1.1971	0.025*	
C314	−0.0615 (2)	0.67420 (13)	1.10544 (15)	0.0243 (5)	
H31B	−0.1083	0.6484	1.1303	0.029*	
C315	−0.1037 (2)	0.65925 (13)	1.02965 (16)	0.0262 (5)	
H31C	−0.1799	0.6235	1.0027	0.031*	
C316	−0.0347 (2)	0.69640 (13)	0.99276 (14)	0.0210 (4)	
H31D	−0.0641	0.6856	0.9406	0.025*	
C317	0.14923 (19)	0.78890 (13)	0.98994 (12)	0.0170 (4)	
H31F	0.1488	0.8447	1.0081	0.020*	
H31E	0.1059	0.7670	0.9362	0.020*	
C321	0.29161 (18)	0.67630 (11)	0.95580 (11)	0.0135 (4)	
C322	0.31064 (19)	0.64179 (12)	0.88477 (12)	0.0160 (4)	
H32	0.3380	0.6734	0.8580	0.019*	
C323	0.2896 (2)	0.56132 (13)	0.85327 (13)	0.0191 (4)	
H32A	0.3011	0.5381	0.8046	0.023*	
C324	0.2520 (2)	0.51488 (13)	0.89280 (13)	0.0208 (4)	
H32B	0.2388	0.4600	0.8714	0.025*	
C325	0.2336 (2)	0.54840 (13)	0.96345 (13)	0.0197 (4)	
H32C	0.2080	0.5165	0.9904	0.024*	
C326	0.25271 (19)	0.62869 (12)	0.99487 (12)	0.0159 (4)	
H32D	0.2393	0.6514	1.0431	0.019*	
C331	0.3570 (2)	0.82443 (12)	0.93283 (11)	0.0146 (4)	
C332	0.2896 (2)	0.80125 (13)	0.85824 (12)	0.0197 (4)	
H33	0.2155	0.7620	0.8406	0.024*	
C333	0.3310 (2)	0.83555 (14)	0.81013 (13)	0.0242 (5)	
H33A	0.2861	0.8187	0.7593	0.029*	
C334	0.4375 (2)	0.89418 (14)	0.83576 (14)	0.0243 (5)	
H33B	0.4651	0.9176	0.8026	0.029*	
C335	0.5037 (2)	0.91855 (13)	0.90961 (13)	0.0214 (4)	
H33C	0.5762	0.9591	0.9273	0.026*	
C336	0.4639 (2)	0.88362 (12)	0.95798 (12)	0.0169 (4)	
H33D	0.5099	0.9002	1.0085	0.020*	
C411	0.77288 (19)	0.88269 (12)	1.12318 (12)	0.0157 (4)	
C412	0.72357 (19)	0.94544 (12)	1.11816 (12)	0.0167 (4)	
H41	0.6552	0.9547	1.1383	0.020*	
C413	0.7734 (2)	0.99428 (13)	1.08405 (14)	0.0231 (5)	
H41A	0.7383	1.0362	1.0802	0.028*	
C414	0.8743 (3)	0.98196 (16)	1.05555 (19)	0.0355 (6)	
H41B	0.9082	1.0150	1.0318	0.043*	
C415	0.9255 (3)	0.92084 (17)	1.0620 (2)	0.0404 (8)	
H41C	0.9958	0.9128	1.0436	0.048*	
C416	0.8746 (2)	0.87141 (14)	1.09529 (16)	0.0275 (5)	
H41D	0.9098	0.8295	1.0989	0.033*	
C417	0.72126 (18)	0.82813 (12)	1.15978 (12)	0.0149 (4)	
H41F	0.7238	0.8595	1.2119	0.018*	
H41E	0.7765	0.7927	1.1624	0.018*	
C421	0.54231 (19)	0.70687 (12)	1.17084 (11)	0.0143 (4)	
C422	0.6373 (2)	0.67576 (12)	1.19754 (12)	0.0174 (4)	
H42	0.7153	0.6879	1.1876	0.021*	
C423	0.6181 (2)	0.62729 (13)	1.23853 (12)	0.0196 (4)	
H42A	0.6833	0.6071	1.2572	0.024*	
C424	0.5041 (2)	0.60853 (13)	1.25217 (13)	0.0216 (4)	
H42B	0.4909	0.5751	1.2798	0.026*	
C425	0.4087 (2)	0.63845 (13)	1.22554 (13)	0.0204 (4)	
H42C	0.3303	0.6249	1.2344	0.024*	
C426	0.4283 (2)	0.68829 (13)	1.18575 (12)	0.0171 (4)	
H42D	0.3636	0.7097	1.1687	0.021*	
C431	0.57838 (18)	0.70326 (12)	1.02637 (11)	0.0137 (4)	
C432	0.55661 (19)	0.62287 (12)	1.00940 (12)	0.0169 (4)	
H43	0.5323	0.6009	1.0442	0.020*	
C433	0.5703 (2)	0.57502 (13)	0.94190 (13)	0.0206 (4)	
H43A	0.5544	0.5204	0.9305	0.025*	
C434	0.6071 (2)	0.60651 (14)	0.89103 (13)	0.0237 (5)	
H43B	0.6167	0.5735	0.8450	0.028*	
C435	0.6299 (2)	0.68622 (14)	0.90744 (13)	0.0231 (5)	
H43C	0.6557	0.7078	0.8727	0.028*	
C436	0.6151 (2)	0.73483 (13)	0.97460 (12)	0.0182 (4)	
H43D	0.6299	0.7894	0.9853	0.022*	
Cl8A	0.0990 (5)	1.1023 (2)	0.24154 (15)	0.0967 (9)	0.413 (2)
Cl7	0.11313 (11)	1.18247 (7)	0.14127 (7)	0.0696 (3)	
Cl8B	0.2143 (3)	1.12252 (14)	0.25605 (10)	0.0967 (9)	0.587 (2)
C2	0.1885 (5)	1.1177 (3)	0.1689 (3)	0.0803 (16)	
H2AB	0.2769	1.1412	0.1923	0.096*	0.413 (2)
H2AA	0.1782	1.0688	0.1268	0.096*	0.413 (2)
H2BC	0.1405	1.0645	0.1376	0.096*	0.587 (2)
H2BD	0.2688	1.1240	0.1560	0.096*	0.587 (2)
Pt1	−0.034074 (7)	0.681952 (4)	0.411236 (4)	0.01360 (2)	
Cl1	−0.05791 (5)	0.62306 (3)	0.28114 (3)	0.02298 (11)	
Cl2	−0.21334 (5)	0.58974 (3)	0.39871 (3)	0.02022 (10)	
P1	0.15125 (5)	0.75067 (3)	0.41467 (3)	0.01512 (10)	
P2	−0.04173 (5)	0.74042 (3)	0.52993 (3)	0.01434 (10)	
C111	0.2781 (2)	0.62945 (13)	0.39782 (14)	0.0221 (5)	
C112	0.1958 (2)	0.56482 (13)	0.39696 (14)	0.0233 (5)	
H11	0.1121	0.5538	0.3712	0.028*	
C113	0.2349 (2)	0.51629 (15)	0.43345 (16)	0.0300 (5)	
H11A	0.1780	0.4725	0.4328	0.036*	
C114	0.3570 (3)	0.53179 (17)	0.4708 (2)	0.0416 (7)	
H11B	0.3837	0.4993	0.4967	0.050*	
C115	0.4403 (3)	0.59498 (18)	0.4704 (2)	0.0448 (8)	
H11C	0.5243	0.6049	0.4951	0.054*	
C116	0.4014 (2)	0.64370 (15)	0.43417 (17)	0.0312 (6)	
H11D	0.4588	0.6868	0.4341	0.037*	
C117	0.2362 (2)	0.68539 (13)	0.36231 (13)	0.0200 (4)	
H11E	0.1832	0.6550	0.3116	0.024*	
H11F	0.3091	0.7177	0.3564	0.024*	
C121	0.26497 (19)	0.80155 (13)	0.50346 (12)	0.0169 (4)	
C122	0.2966 (2)	0.75976 (14)	0.55022 (13)	0.0220 (4)	
H12	0.2523	0.7077	0.5379	0.026*	
C123	0.3921 (2)	0.79383 (16)	0.61437 (14)	0.0271 (5)	
H12A	0.4132	0.7649	0.6455	0.032*	
C124	0.4567 (2)	0.86991 (17)	0.63316 (14)	0.0283 (5)	
H12B	0.5226	0.8930	0.6768	0.034*	
C125	0.4248 (2)	0.91219 (15)	0.58819 (14)	0.0248 (5)	
H12C	0.4684	0.9646	0.6015	0.030*	
C126	0.3296 (2)	0.87859 (13)	0.52366 (13)	0.0197 (4)	
H12D	0.3085	0.9081	0.4932	0.024*	
C131	0.1427 (2)	0.81863 (12)	0.36458 (12)	0.0167 (4)	
C132	0.0304 (2)	0.83561 (13)	0.34178 (12)	0.0182 (4)	
H13	−0.0403	0.8132	0.3540	0.022*	
C133	0.0220 (2)	0.88532 (13)	0.30106 (13)	0.0215 (4)	
H13A	−0.0543	0.8972	0.2861	0.026*	
C134	0.1248 (2)	0.91756 (14)	0.28222 (13)	0.0238 (5)	
H13B	0.1185	0.9512	0.2542	0.029*	
C135	0.2368 (2)	0.90074 (14)	0.30427 (13)	0.0224 (5)	
H13C	0.3071	0.9228	0.2914	0.027*	
C136	0.2457 (2)	0.85163 (13)	0.34508 (12)	0.0195 (4)	
H13D	0.3224	0.8402	0.3600	0.023*	
C211	−0.25526 (19)	0.79796 (13)	0.50304 (13)	0.0176 (4)	
C212	−0.2580 (2)	0.87310 (14)	0.54565 (13)	0.0229 (5)	
H21	−0.2222	0.8928	0.5981	0.027*	
C213	−0.3129 (2)	0.91931 (15)	0.51203 (15)	0.0272 (5)	
H21A	−0.3142	0.9704	0.5415	0.033*	
C214	−0.3656 (2)	0.89102 (15)	0.43559 (15)	0.0257 (5)	
H21B	−0.4039	0.9224	0.4128	0.031*	
C215	−0.3624 (2)	0.81671 (14)	0.39252 (14)	0.0248 (5)	
H21C	−0.3977	0.7974	0.3401	0.030*	
C216	−0.3076 (2)	0.77037 (13)	0.42605 (13)	0.0209 (4)	
H21D	−0.3057	0.7195	0.3963	0.025*	
C217	−0.1977 (2)	0.74752 (13)	0.54004 (12)	0.0176 (4)	
H21E	−0.2516	0.6945	0.5189	0.021*	
H21F	−0.1947	0.7680	0.5941	0.021*	
C221	0.0035 (2)	0.68681 (13)	0.59019 (13)	0.0188 (4)	
C222	0.0541 (2)	0.62382 (14)	0.56271 (14)	0.0224 (5)	
H22	0.0640	0.6096	0.5130	0.027*	
C223	0.0904 (2)	0.58132 (15)	0.60731 (16)	0.0286 (5)	
H22A	0.1260	0.5389	0.5883	0.034*	
C224	0.0741 (2)	0.60135 (16)	0.67949 (16)	0.0311 (6)	
H22B	0.0992	0.5728	0.7101	0.037*	
C225	0.0216 (2)	0.66279 (16)	0.70709 (14)	0.0292 (5)	
H22C	0.0097	0.6757	0.7564	0.035*	
C226	−0.0141 (2)	0.70577 (14)	0.66287 (13)	0.0236 (5)	
H22D	−0.0502	0.7479	0.6820	0.028*	
C231	0.04475 (19)	0.84080 (12)	0.57503 (12)	0.0152 (4)	
C232	0.03151 (19)	0.89170 (12)	0.53567 (12)	0.0171 (4)	
H23	−0.0233	0.8737	0.4874	0.020*	
C233	0.0981 (2)	0.96848 (13)	0.56689 (13)	0.0196 (4)	
H23A	0.0890	1.0028	0.5400	0.023*	
C234	0.1780 (2)	0.99501 (13)	0.63740 (13)	0.0211 (4)	
H23B	0.2243	1.0474	0.6584	0.025*	
C235	0.1902 (2)	0.94532 (14)	0.67714 (13)	0.0222 (5)	
H23C	0.2441	0.9639	0.7257	0.027*	
C236	0.1241 (2)	0.86841 (14)	0.64629 (12)	0.0205 (4)	
H23D	0.1330	0.8345	0.6738	0.025*	
Cl	0.65614 (15)	0.66456 (15)	0.68851 (11)	0.1016 (7)	0.754 (3)
Cl9B	0.4093 (3)	0.6313 (3)	0.7090 (2)	0.0673 (8)	0.561 (5)
Cl9A	0.4227 (4)	0.6689 (3)	0.7294 (3)	0.0673 (8)	0.439 (5)
Cl0A	0.5980 (5)	0.5947 (5)	0.6825 (4)	0.1016 (7)	0.246 (3)
C3	0.4945 (8)	0.5929 (4)	0.6778 (4)	0.147 (4)	
H	0.4492	0.5477	0.6367	0.177*	0.246 (3)
H3A	0.4484	0.5661	0.6248	0.177*	0.754 (3)
H3B	0.5051	0.5543	0.7024	0.177*	0.754 (3)

### Atomic displacement parameters (Å^2^)

**Table:**

	*U*^11^	*U*^22^	*U*^33^	*U*^12^	*U*^13^	*U*^23^
Cl5	0.0373 (4)	0.0380 (4)	0.0509 (4)	0.0072 (3)	0.0125 (3)	0.0210 (3)
Cl6	0.0305 (3)	0.0579 (4)	0.0267 (3)	0.0136 (3)	0.0095 (3)	0.0164 (3)
C1	0.0224 (12)	0.0274 (12)	0.0371 (14)	0.0071 (10)	0.0129 (11)	0.0069 (11)
Pt2	0.01004 (4)	0.01054 (3)	0.01013 (4)	0.00232 (3)	0.00229 (3)	0.00296 (3)
Cl3	0.0159 (2)	0.0254 (3)	0.0127 (2)	0.00461 (19)	0.00083 (18)	−0.00128 (19)
Cl4	0.0198 (2)	0.0135 (2)	0.0222 (2)	0.00696 (18)	0.00808 (19)	0.00722 (18)
P3	0.0119 (2)	0.0121 (2)	0.0111 (2)	0.00317 (18)	0.00223 (18)	0.00439 (18)
P4	0.0107 (2)	0.0131 (2)	0.0112 (2)	0.00354 (18)	0.00258 (18)	0.00418 (18)
C311	0.0134 (9)	0.0158 (9)	0.0191 (10)	0.0060 (7)	0.0047 (8)	0.0037 (8)
C312	0.0145 (9)	0.0166 (9)	0.0180 (10)	0.0019 (8)	0.0044 (8)	0.0032 (8)
C313	0.0227 (11)	0.0180 (10)	0.0231 (11)	0.0057 (8)	0.0100 (9)	0.0073 (9)
C314	0.0207 (11)	0.0171 (10)	0.0394 (14)	0.0043 (8)	0.0145 (10)	0.0120 (10)
C315	0.0140 (10)	0.0163 (10)	0.0429 (15)	0.0001 (8)	0.0022 (10)	0.0077 (10)
C316	0.0162 (10)	0.0181 (10)	0.0234 (11)	0.0049 (8)	0.0003 (9)	0.0022 (8)
C317	0.0141 (9)	0.0221 (10)	0.0151 (10)	0.0067 (8)	0.0019 (8)	0.0068 (8)
C321	0.0108 (9)	0.0127 (9)	0.0147 (9)	0.0011 (7)	0.0010 (7)	0.0040 (7)
C322	0.0159 (9)	0.0161 (9)	0.0147 (9)	0.0027 (7)	0.0026 (8)	0.0047 (8)
C323	0.0195 (10)	0.0172 (10)	0.0174 (10)	0.0035 (8)	0.0038 (8)	0.0025 (8)
C324	0.0190 (10)	0.0128 (9)	0.0262 (12)	0.0009 (8)	0.0039 (9)	0.0032 (8)
C325	0.0175 (10)	0.0165 (10)	0.0263 (11)	0.0018 (8)	0.0068 (9)	0.0096 (9)
C326	0.0151 (9)	0.0162 (9)	0.0156 (10)	0.0027 (7)	0.0035 (8)	0.0052 (8)
C331	0.0187 (10)	0.0146 (9)	0.0131 (9)	0.0067 (8)	0.0044 (8)	0.0068 (7)
C332	0.0224 (11)	0.0199 (10)	0.0168 (10)	0.0065 (8)	0.0019 (8)	0.0072 (8)
C333	0.0339 (13)	0.0284 (12)	0.0157 (10)	0.0143 (10)	0.0068 (9)	0.0112 (9)
C334	0.0350 (13)	0.0258 (11)	0.0230 (11)	0.0138 (10)	0.0141 (10)	0.0159 (10)
C335	0.0261 (11)	0.0192 (10)	0.0234 (11)	0.0054 (9)	0.0106 (9)	0.0109 (9)
C336	0.0202 (10)	0.0149 (9)	0.0168 (10)	0.0043 (8)	0.0053 (8)	0.0069 (8)
C411	0.0139 (9)	0.0145 (9)	0.0163 (10)	0.0021 (7)	0.0028 (8)	0.0032 (8)
C412	0.0141 (9)	0.0151 (9)	0.0183 (10)	0.0032 (7)	0.0033 (8)	0.0028 (8)
C413	0.0244 (11)	0.0163 (10)	0.0314 (13)	0.0071 (9)	0.0093 (10)	0.0098 (9)
C414	0.0393 (15)	0.0277 (13)	0.0584 (19)	0.0147 (11)	0.0314 (14)	0.0266 (13)
C415	0.0407 (16)	0.0358 (15)	0.071 (2)	0.0222 (13)	0.0421 (16)	0.0325 (15)
C416	0.0263 (12)	0.0217 (11)	0.0458 (15)	0.0135 (9)	0.0205 (11)	0.0168 (11)
C417	0.0113 (9)	0.0156 (9)	0.0149 (9)	0.0016 (7)	0.0010 (7)	0.0036 (7)
C421	0.0164 (9)	0.0149 (9)	0.0112 (9)	0.0039 (7)	0.0027 (7)	0.0041 (7)
C422	0.0179 (10)	0.0182 (10)	0.0173 (10)	0.0070 (8)	0.0053 (8)	0.0060 (8)
C423	0.0257 (11)	0.0182 (10)	0.0156 (10)	0.0087 (8)	0.0028 (8)	0.0064 (8)
C424	0.0325 (12)	0.0179 (10)	0.0158 (10)	0.0055 (9)	0.0070 (9)	0.0076 (8)
C425	0.0213 (11)	0.0213 (10)	0.0185 (10)	0.0018 (8)	0.0084 (9)	0.0067 (8)
C426	0.0170 (10)	0.0198 (10)	0.0140 (9)	0.0043 (8)	0.0032 (8)	0.0055 (8)
C431	0.0111 (9)	0.0162 (9)	0.0133 (9)	0.0043 (7)	0.0029 (7)	0.0040 (7)
C432	0.0147 (9)	0.0178 (10)	0.0176 (10)	0.0044 (8)	0.0029 (8)	0.0054 (8)
C433	0.0182 (10)	0.0169 (10)	0.0213 (11)	0.0047 (8)	0.0027 (8)	0.0003 (8)
C434	0.0187 (11)	0.0272 (12)	0.0179 (11)	0.0036 (9)	0.0062 (9)	−0.0021 (9)
C435	0.0203 (11)	0.0298 (12)	0.0172 (10)	0.0015 (9)	0.0078 (9)	0.0060 (9)
C436	0.0174 (10)	0.0195 (10)	0.0164 (10)	0.0017 (8)	0.0045 (8)	0.0057 (8)
Cl8A	0.207 (3)	0.0791 (12)	0.0369 (7)	0.0955 (19)	0.0372 (13)	0.0302 (8)
Cl7	0.0767 (7)	0.0626 (6)	0.0701 (7)	0.0178 (5)	−0.0046 (5)	0.0361 (5)
Cl8B	0.207 (3)	0.0791 (12)	0.0369 (7)	0.0955 (19)	0.0372 (13)	0.0302 (8)
C2	0.113 (4)	0.085 (3)	0.056 (3)	0.072 (3)	0.014 (3)	0.022 (2)
Pt1	0.01359 (4)	0.01389 (4)	0.01175 (4)	0.00352 (3)	0.00174 (3)	0.00298 (3)
Cl1	0.0216 (3)	0.0271 (3)	0.0138 (2)	0.0058 (2)	0.00265 (19)	−0.0008 (2)
Cl2	0.0186 (2)	0.0168 (2)	0.0228 (3)	0.00122 (18)	0.0014 (2)	0.00712 (19)
P1	0.0150 (2)	0.0172 (2)	0.0131 (2)	0.00517 (19)	0.00369 (19)	0.00440 (19)
P2	0.0143 (2)	0.0159 (2)	0.0122 (2)	0.00280 (19)	0.00229 (19)	0.00495 (19)
C111	0.0201 (11)	0.0206 (10)	0.0259 (12)	0.0094 (9)	0.0091 (9)	0.0043 (9)
C112	0.0187 (11)	0.0203 (10)	0.0272 (12)	0.0055 (9)	0.0027 (9)	0.0043 (9)
C113	0.0253 (12)	0.0202 (11)	0.0426 (15)	0.0061 (9)	0.0052 (11)	0.0099 (11)
C114	0.0301 (14)	0.0297 (14)	0.067 (2)	0.0127 (12)	0.0005 (14)	0.0232 (14)
C115	0.0190 (12)	0.0343 (15)	0.078 (2)	0.0090 (11)	−0.0032 (14)	0.0231 (16)
C116	0.0185 (11)	0.0234 (12)	0.0517 (17)	0.0071 (9)	0.0078 (11)	0.0128 (11)
C117	0.0180 (10)	0.0207 (10)	0.0215 (11)	0.0066 (8)	0.0085 (9)	0.0044 (8)
C121	0.0139 (9)	0.0225 (10)	0.0136 (9)	0.0065 (8)	0.0036 (8)	0.0043 (8)
C122	0.0204 (11)	0.0277 (11)	0.0209 (11)	0.0079 (9)	0.0064 (9)	0.0108 (9)
C123	0.0238 (12)	0.0421 (14)	0.0202 (11)	0.0140 (11)	0.0051 (9)	0.0146 (10)
C124	0.0191 (11)	0.0427 (15)	0.0170 (11)	0.0071 (10)	0.0005 (9)	0.0041 (10)
C125	0.0188 (11)	0.0282 (12)	0.0206 (11)	0.0016 (9)	0.0026 (9)	0.0023 (9)
C126	0.0170 (10)	0.0224 (10)	0.0186 (10)	0.0049 (8)	0.0055 (8)	0.0050 (8)
C131	0.0189 (10)	0.0174 (9)	0.0125 (9)	0.0043 (8)	0.0029 (8)	0.0038 (8)
C132	0.0182 (10)	0.0215 (10)	0.0135 (9)	0.0053 (8)	0.0043 (8)	0.0037 (8)
C133	0.0243 (11)	0.0227 (11)	0.0171 (10)	0.0093 (9)	0.0037 (9)	0.0053 (8)
C134	0.0331 (13)	0.0212 (11)	0.0178 (11)	0.0066 (9)	0.0060 (9)	0.0079 (9)
C135	0.0247 (11)	0.0219 (11)	0.0196 (11)	0.0017 (9)	0.0078 (9)	0.0064 (9)
C136	0.0173 (10)	0.0213 (10)	0.0181 (10)	0.0043 (8)	0.0037 (8)	0.0051 (8)
C211	0.0131 (9)	0.0217 (10)	0.0196 (10)	0.0041 (8)	0.0056 (8)	0.0084 (8)
C212	0.0251 (11)	0.0257 (11)	0.0177 (10)	0.0079 (9)	0.0078 (9)	0.0049 (9)
C213	0.0333 (13)	0.0235 (11)	0.0282 (13)	0.0122 (10)	0.0136 (11)	0.0074 (10)
C214	0.0260 (12)	0.0269 (12)	0.0303 (13)	0.0102 (10)	0.0086 (10)	0.0153 (10)
C215	0.0259 (12)	0.0266 (12)	0.0196 (11)	0.0059 (9)	0.0004 (9)	0.0081 (9)
C216	0.0206 (11)	0.0197 (10)	0.0194 (11)	0.0039 (8)	0.0020 (9)	0.0046 (8)
C217	0.0157 (10)	0.0220 (10)	0.0154 (10)	0.0027 (8)	0.0054 (8)	0.0069 (8)
C221	0.0165 (10)	0.0204 (10)	0.0184 (10)	0.0010 (8)	0.0002 (8)	0.0095 (8)
C222	0.0180 (10)	0.0232 (11)	0.0255 (12)	0.0024 (9)	0.0014 (9)	0.0113 (9)
C223	0.0221 (12)	0.0256 (12)	0.0401 (15)	0.0035 (9)	0.0010 (10)	0.0189 (11)
C224	0.0258 (12)	0.0322 (13)	0.0343 (14)	−0.0034 (10)	−0.0055 (10)	0.0226 (11)
C225	0.0292 (13)	0.0332 (13)	0.0212 (12)	−0.0050 (10)	−0.0023 (10)	0.0152 (10)
C226	0.0247 (11)	0.0258 (11)	0.0184 (11)	0.0001 (9)	0.0023 (9)	0.0099 (9)
C231	0.0131 (9)	0.0168 (9)	0.0137 (9)	0.0029 (7)	0.0033 (7)	0.0033 (7)
C232	0.0148 (9)	0.0192 (10)	0.0157 (10)	0.0038 (8)	0.0028 (8)	0.0046 (8)
C233	0.0174 (10)	0.0184 (10)	0.0221 (11)	0.0045 (8)	0.0035 (8)	0.0066 (8)
C234	0.0169 (10)	0.0191 (10)	0.0212 (11)	0.0024 (8)	0.0041 (8)	0.0000 (8)
C235	0.0208 (11)	0.0267 (11)	0.0130 (10)	0.0025 (9)	0.0008 (8)	0.0019 (8)
C236	0.0205 (11)	0.0251 (11)	0.0148 (10)	0.0035 (9)	0.0033 (8)	0.0069 (8)
Cl	0.0465 (8)	0.1474 (19)	0.0832 (11)	0.0166 (9)	0.0206 (8)	0.0033 (12)
Cl9B	0.0537 (10)	0.098 (2)	0.0632 (18)	0.0041 (17)	0.0170 (12)	0.0516 (18)
Cl9A	0.0537 (10)	0.098 (2)	0.0632 (18)	0.0041 (17)	0.0170 (12)	0.0516 (18)
Cl0A	0.0465 (8)	0.1474 (19)	0.0832 (11)	0.0166 (9)	0.0206 (8)	0.0033 (12)
C3	0.253 (12)	0.063 (4)	0.069 (4)	0.006 (5)	−0.015 (6)	−0.009 (3)

### Geometric parameters (Å, °)

**Table:**

Cl5—C1	1.756 (3)	P3—C317	1.845 (2)
Cl6—C1	1.767 (3)	P4—C421	1.818 (2)
C1—H1A	0.9900	P4—C431	1.820 (2)
C1—H1B	0.9900	P4—C417	1.848 (2)
Pt1—P2	2.2436 (6)	C311—C316	1.393 (3)
Pt1—P1	2.2630 (6)	C311—C312	1.401 (3)
Pt1—Cl1	2.3602 (6)	C311—C317	1.510 (3)
Pt1—Cl2	2.3663 (5)	C312—C313	1.392 (3)
P1—C121	1.822 (2)	C312—H31	0.9500
P1—C131	1.822 (2)	C313—C314	1.385 (3)
P1—C117	1.850 (2)	C313—H31A	0.9500
P2—C231	1.816 (2)	C314—C315	1.383 (4)
P2—C221	1.817 (2)	C314—H31B	0.9500
P2—C217	1.846 (2)	C315—C316	1.393 (3)
C111—C112	1.394 (3)	C315—H31C	0.9500
C111—C116	1.395 (3)	C316—H31D	0.9500
C111—C117	1.509 (3)	C317—H31F	0.9900
C112—C113	1.389 (4)	C317—H31E	0.9900
C112—H11	0.9500	C321—C322	1.400 (3)
C113—C114	1.387 (4)	C321—C326	1.402 (3)
C113—H11A	0.9500	C322—C323	1.392 (3)
C114—C115	1.389 (4)	C322—H32	0.9500
C114—H11B	0.9500	C323—C324	1.388 (3)
C115—C116	1.389 (4)	C323—H32A	0.9500
C115—H11C	0.9500	C324—C325	1.387 (3)
C116—H11D	0.9500	C324—H32B	0.9500
C117—H11E	0.9900	C325—C326	1.390 (3)
C117—H11F	0.9900	C325—H32C	0.9500
C121—C126	1.398 (3)	C326—H32D	0.9500
C121—C122	1.403 (3)	C331—C336	1.396 (3)
C122—C123	1.389 (3)	C331—C332	1.403 (3)
C122—H12	0.9500	C332—C333	1.390 (3)
C123—C124	1.386 (4)	C332—H33	0.9500
C123—H12A	0.9500	C333—C334	1.387 (4)
C124—C125	1.384 (4)	C333—H33A	0.9500
C124—H12B	0.9500	C334—C335	1.385 (3)
C125—C126	1.391 (3)	C334—H33B	0.9500
C125—H12C	0.9500	C335—C336	1.394 (3)
C126—H12D	0.9500	C335—H33C	0.9500
C131—C132	1.396 (3)	C336—H33D	0.9500
C131—C136	1.404 (3)	C411—C416	1.386 (3)
C132—C133	1.394 (3)	C411—C412	1.399 (3)
C132—H13	0.9500	C411—C417	1.511 (3)
C133—C134	1.389 (3)	C412—C413	1.388 (3)
C133—H13A	0.9500	C412—H41	0.9500
C134—C135	1.389 (4)	C413—C414	1.386 (4)
C134—H13B	0.9500	C413—H41A	0.9500
C135—C136	1.386 (3)	C414—C415	1.390 (4)
C135—H13C	0.9500	C414—H41B	0.9500
C136—H13D	0.9500	C415—C416	1.389 (4)
C211—C212	1.395 (3)	C415—H41C	0.9500
C211—C216	1.395 (3)	C416—H41D	0.9500
C211—C217	1.509 (3)	C417—H41F	0.9900
C212—C213	1.390 (4)	C417—H41E	0.9900
C212—H21	0.9500	C421—C426	1.395 (3)
C213—C214	1.387 (4)	C421—C422	1.402 (3)
C213—H21A	0.9500	C422—C423	1.391 (3)
C214—C215	1.387 (3)	C422—H42	0.9500
C214—H21B	0.9500	C423—C424	1.385 (3)
C215—C216	1.391 (3)	C423—H42A	0.9500
C215—H21C	0.9500	C424—C425	1.391 (3)
C216—H21D	0.9500	C424—H42B	0.9500
C217—H21E	0.9900	C425—C426	1.394 (3)
C217—H21F	0.9900	C425—H42C	0.9500
C221—C222	1.392 (3)	C426—H42D	0.9500
C221—C226	1.402 (3)	C431—C432	1.398 (3)
C222—C223	1.395 (3)	C431—C436	1.402 (3)
C222—H22	0.9500	C432—C433	1.388 (3)
C223—C224	1.387 (4)	C432—H43	0.9500
C223—H22A	0.9500	C433—C434	1.385 (3)
C224—C225	1.384 (4)	C433—H43A	0.9500
C224—H22B	0.9500	C434—C435	1.386 (3)
C225—C226	1.395 (3)	C434—H43B	0.9500
C225—H22C	0.9500	C435—C436	1.393 (3)
C226—H22D	0.9500	C435—H43C	0.9500
C231—C236	1.395 (3)	C436—H43D	0.9500
C231—C232	1.399 (3)	Cl8A—C2	1.945 (7)
C232—C233	1.388 (3)	Cl7—C2	1.747 (4)
C232—H23	0.9500	Cl8B—C2	1.632 (5)
C233—C234	1.387 (3)	C2—H2AB	0.9900
C233—H23A	0.9500	C2—H2AA	0.9900
C234—C235	1.384 (3)	C2—H2BC	0.9900
C234—H23B	0.9500	C2—H2BD	0.9900
C235—C236	1.388 (3)	Cl—C3	2.026 (8)
C235—H23C	0.9500	Cl9B—C3	1.412 (8)
C236—H23D	0.9500	Cl9B—Cl0A	2.455 (7)
Pt2—P3	2.2505 (5)	Cl9A—C3	1.862 (9)
Pt2—P4	2.2505 (5)	Cl0A—C3	1.162 (8)
Pt2—Cl3	2.3531 (5)	C3—H	0.9500
Pt2—Cl4	2.3713 (5)	C3—H3A	0.9900
P3—C331	1.814 (2)	C3—H3B	0.9900
P3—C321	1.825 (2)		
			
Cl5—C1—Cl6	111.73 (14)	C421—P4—Pt2	112.41 (7)
Cl5—C1—H1A	109.3	C431—P4—Pt2	120.23 (7)
Cl6—C1—H1A	109.3	C417—P4—Pt2	111.31 (7)
Cl5—C1—H1B	109.3	C316—C311—C312	118.4 (2)
Cl6—C1—H1B	109.3	C316—C311—C317	119.4 (2)
H1A—C1—H1B	107.9	C312—C311—C317	122.28 (19)
P2—Pt1—P1	99.22 (2)	C313—C312—C311	120.8 (2)
P2—Pt1—Cl1	170.89 (2)	C313—C312—H31	119.6
P1—Pt1—Cl1	86.95 (2)	C311—C312—H31	119.6
P2—Pt1—Cl2	87.33 (2)	C314—C313—C312	119.9 (2)
P1—Pt1—Cl2	169.39 (2)	C314—C313—H31A	120.0
Cl1—Pt1—Cl2	87.59 (2)	C312—C313—H31A	120.0
C121—P1—C131	107.25 (10)	C315—C314—C313	120.0 (2)
C121—P1—C117	101.08 (10)	C315—C314—H31B	120.0
C131—P1—C117	101.70 (10)	C313—C314—H31B	120.0
C121—P1—Pt1	120.48 (7)	C314—C315—C316	120.2 (2)
C131—P1—Pt1	113.76 (7)	C314—C315—H31C	119.9
C117—P1—Pt1	110.26 (8)	C316—C315—H31C	119.9
C231—P2—C221	108.18 (10)	C311—C316—C315	120.7 (2)
C231—P2—C217	102.33 (10)	C311—C316—H31D	119.7
C221—P2—C217	102.96 (10)	C315—C316—H31D	119.7
C231—P2—Pt1	115.95 (7)	C311—C317—P3	116.36 (15)
C221—P2—Pt1	112.65 (8)	C311—C317—H31F	108.2
C217—P2—Pt1	113.53 (7)	P3—C317—H31F	108.2
C112—C111—C116	119.1 (2)	C311—C317—H31E	108.2
C112—C111—C117	121.4 (2)	P3—C317—H31E	108.2
C116—C111—C117	119.4 (2)	H31F—C317—H31E	107.4
C113—C112—C111	120.6 (2)	C322—C321—C326	119.04 (19)
C113—C112—H11	119.7	C322—C321—P3	122.43 (16)
C111—C112—H11	119.7	C326—C321—P3	118.49 (16)
C114—C113—C112	119.9 (2)	C323—C322—C321	120.3 (2)
C114—C113—H11A	120.0	C323—C322—H32	119.9
C112—C113—H11A	120.0	C321—C322—H32	119.9
C113—C114—C115	119.8 (3)	C324—C323—C322	120.1 (2)
C113—C114—H11B	120.1	C324—C323—H32A	119.9
C115—C114—H11B	120.1	C322—C323—H32A	119.9
C116—C115—C114	120.3 (3)	C325—C324—C323	120.2 (2)
C116—C115—H11C	119.8	C325—C324—H32B	119.9
C114—C115—H11C	119.8	C323—C324—H32B	119.9
C115—C116—C111	120.2 (2)	C324—C325—C326	120.1 (2)
C115—C116—H11D	119.9	C324—C325—H32C	120.0
C111—C116—H11D	119.9	C326—C325—H32C	120.0
C111—C117—P1	115.25 (16)	C325—C326—C321	120.3 (2)
C111—C117—H11E	108.5	C325—C326—H32D	119.8
P1—C117—H11E	108.5	C321—C326—H32D	119.8
C111—C117—H11F	108.5	C336—C331—C332	118.88 (19)
P1—C117—H11F	108.5	C336—C331—P3	118.63 (16)
H11E—C117—H11F	107.5	C332—C331—P3	122.48 (17)
C126—C121—C122	118.7 (2)	C333—C332—C331	120.1 (2)
C126—C121—P1	122.45 (17)	C333—C332—H33	120.0
C122—C121—P1	118.61 (17)	C331—C332—H33	120.0
C123—C122—C121	120.5 (2)	C334—C333—C332	120.4 (2)
C123—C122—H12	119.7	C334—C333—H33A	119.8
C121—C122—H12	119.7	C332—C333—H33A	119.8
C124—C123—C122	120.2 (2)	C335—C334—C333	120.0 (2)
C124—C123—H12A	119.9	C335—C334—H33B	120.0
C122—C123—H12A	119.9	C333—C334—H33B	120.0
C125—C124—C123	119.8 (2)	C334—C335—C336	120.0 (2)
C125—C124—H12B	120.1	C334—C335—H33C	120.0
C123—C124—H12B	120.1	C336—C335—H33C	120.0
C124—C125—C126	120.5 (2)	C335—C336—C331	120.6 (2)
C124—C125—H12C	119.7	C335—C336—H33D	119.7
C126—C125—H12C	119.7	C331—C336—H33D	119.7
C125—C126—C121	120.3 (2)	C416—C411—C412	118.7 (2)
C125—C126—H12D	119.9	C416—C411—C417	118.89 (19)
C121—C126—H12D	119.9	C412—C411—C417	122.35 (19)
C132—C131—C136	118.9 (2)	C413—C412—C411	120.7 (2)
C132—C131—P1	119.70 (17)	C413—C412—H41	119.6
C136—C131—P1	121.28 (17)	C411—C412—H41	119.6
C133—C132—C131	120.1 (2)	C414—C413—C412	120.1 (2)
C133—C132—H13	119.9	C414—C413—H41A	120.0
C131—C132—H13	119.9	C412—C413—H41A	120.0
C134—C133—C132	120.3 (2)	C413—C414—C415	119.4 (2)
C134—C133—H13A	119.9	C413—C414—H41B	120.3
C132—C133—H13A	119.9	C415—C414—H41B	120.3
C135—C134—C133	120.1 (2)	C416—C415—C414	120.4 (2)
C135—C134—H13B	120.0	C416—C415—H41C	119.8
C133—C134—H13B	120.0	C414—C415—H41C	119.8
C136—C135—C134	119.9 (2)	C411—C416—C415	120.6 (2)
C136—C135—H13C	120.1	C411—C416—H41D	119.7
C134—C135—H13C	120.1	C415—C416—H41D	119.7
C135—C136—C131	120.7 (2)	C411—C417—P4	117.30 (15)
C135—C136—H13D	119.6	C411—C417—H41F	108.0
C131—C136—H13D	119.6	P4—C417—H41F	108.0
C212—C211—C216	118.8 (2)	C411—C417—H41E	108.0
C212—C211—C217	120.3 (2)	P4—C417—H41E	108.0
C216—C211—C217	120.9 (2)	H41F—C417—H41E	107.2
C213—C212—C211	120.5 (2)	C426—C421—C422	119.02 (19)
C213—C212—H21	119.7	C426—C421—P4	119.61 (16)
C211—C212—H21	119.7	C422—C421—P4	121.34 (16)
C214—C213—C212	120.2 (2)	C423—C422—C421	120.4 (2)
C214—C213—H21A	119.9	C423—C422—H42	119.8
C212—C213—H21A	119.9	C421—C422—H42	119.8
C213—C214—C215	119.8 (2)	C424—C423—C422	120.0 (2)
C213—C214—H21B	120.1	C424—C423—H42A	120.0
C215—C214—H21B	120.1	C422—C423—H42A	120.0
C214—C215—C216	120.1 (2)	C423—C424—C425	120.2 (2)
C214—C215—H21C	120.0	C423—C424—H42B	119.9
C216—C215—H21C	120.0	C425—C424—H42B	119.9
C215—C216—C211	120.6 (2)	C424—C425—C426	119.9 (2)
C215—C216—H21D	119.7	C424—C425—H42C	120.0
C211—C216—H21D	119.7	C426—C425—H42C	120.0
C211—C217—P2	115.62 (15)	C425—C426—C421	120.4 (2)
C211—C217—H21E	108.4	C425—C426—H42D	119.8
P2—C217—H21E	108.4	C421—C426—H42D	119.8
C211—C217—H21F	108.4	C432—C431—C436	119.12 (19)
P2—C217—H21F	108.4	C432—C431—P4	121.60 (16)
H21E—C217—H21F	107.4	C436—C431—P4	119.26 (16)
C222—C221—C226	119.0 (2)	C433—C432—C431	120.3 (2)
C222—C221—P2	119.04 (18)	C433—C432—H43	119.8
C226—C221—P2	121.89 (18)	C431—C432—H43	119.8
C221—C222—C223	120.8 (2)	C434—C433—C432	120.4 (2)
C221—C222—H22	119.6	C434—C433—H43A	119.8
C223—C222—H22	119.6	C432—C433—H43A	119.8
C224—C223—C222	119.6 (3)	C433—C434—C435	119.9 (2)
C224—C223—H22A	120.2	C433—C434—H43B	120.1
C222—C223—H22A	120.2	C435—C434—H43B	120.1
C225—C224—C223	120.3 (2)	C434—C435—C436	120.3 (2)
C225—C224—H22B	119.8	C434—C435—H43C	119.8
C223—C224—H22B	119.8	C436—C435—H43C	119.8
C224—C225—C226	120.3 (2)	C435—C436—C431	120.0 (2)
C224—C225—H22C	119.9	C435—C436—H43D	120.0
C226—C225—H22C	119.9	C431—C436—H43D	120.0
C225—C226—C221	120.0 (2)	Cl8B—C2—Cl7	119.9 (3)
C225—C226—H22D	120.0	Cl7—C2—Cl8A	98.7 (2)
C221—C226—H22D	120.0	Cl8B—C2—H2AB	72.4
C236—C231—C232	119.2 (2)	Cl7—C2—H2AB	112.0
C236—C231—P2	122.74 (17)	Cl8A—C2—H2AB	112.0
C232—C231—P2	118.03 (16)	Cl8B—C2—H2AA	122.6
C233—C232—C231	120.2 (2)	Cl7—C2—H2AA	112.0
C233—C232—H23	119.9	Cl8A—C2—H2AA	112.0
C231—C232—H23	119.9	H2AB—C2—H2AA	109.7
C234—C233—C232	120.0 (2)	Cl8B—C2—H2BC	107.3
C234—C233—H23A	120.0	Cl7—C2—H2BC	107.3
C232—C233—H23A	120.0	Cl8A—C2—H2BC	83.9
C235—C234—C233	120.1 (2)	H2AB—C2—H2BC	134.0
C235—C234—H23B	120.0	Cl8B—C2—H2BD	107.3
C233—C234—H23B	120.0	Cl7—C2—H2BD	107.3
C234—C235—C236	120.3 (2)	Cl8A—C2—H2BD	146.8
C234—C235—H23C	119.9	H2AA—C2—H2BD	77.1
C236—C235—H23C	119.9	H2BC—C2—H2BD	106.9
C235—C236—C231	120.2 (2)	Cl0A—C3—Cl9B	144.9 (7)
C235—C236—H23D	119.9	Cl0A—C3—Cl9A	128.6 (6)
C231—C236—H23D	119.9	Cl9B—C3—Cl	114.3 (4)
P3—Pt2—P4	99.115 (19)	Cl9A—C3—Cl	97.2 (3)
P3—Pt2—Cl3	172.666 (19)	Cl0A—C3—H	107.6
P4—Pt2—Cl3	85.919 (19)	Cl9B—C3—H	107.6
P3—Pt2—Cl4	88.952 (19)	Cl9A—C3—H	123.3
P4—Pt2—Cl4	169.871 (19)	Cl—C3—H	128.7
Cl3—Pt2—Cl4	86.653 (19)	Cl0A—C3—H3A	109.2
C331—P3—C321	107.13 (10)	Cl9B—C3—H3A	101.8
C331—P3—C317	102.76 (10)	Cl9A—C3—H3A	112.3
C321—P3—C317	101.84 (10)	Cl—C3—H3A	112.3
C331—P3—Pt2	112.67 (7)	Cl0A—C3—H3B	79.4
C321—P3—Pt2	118.52 (7)	Cl9B—C3—H3B	105.4
C317—P3—Pt2	112.28 (7)	Cl9A—C3—H3B	112.3
C421—P4—C431	104.93 (9)	Cl—C3—H3B	112.3
C421—P4—C417	103.24 (10)	H—C3—H3B	82.2
C431—P4—C417	103.04 (10)	H3A—C3—H3B	109.9
			
P4—Pt2—P3—C331	−94.06 (8)	Cl2—Pt1—P1—C121	−105.45 (13)
Cl4—Pt2—P3—C331	79.79 (8)	P2—Pt1—P1—C131	−107.28 (8)
P4—Pt2—P3—C321	32.13 (8)	Cl1—Pt1—P1—C131	65.98 (8)
Cl4—Pt2—P3—C321	−154.03 (8)	Cl2—Pt1—P1—C131	125.11 (12)
P4—Pt2—P3—C317	150.49 (8)	P2—Pt1—P1—C117	139.23 (8)
Cl4—Pt2—P3—C317	−35.67 (8)	Cl1—Pt1—P1—C117	−47.51 (8)
P3—Pt2—P4—C421	−108.12 (7)	Cl2—Pt1—P1—C117	11.62 (14)
Cl3—Pt2—P4—C421	66.51 (8)	P1—Pt1—P2—C231	27.49 (8)
Cl4—Pt2—P4—C421	109.44 (12)	Cl2—Pt1—P2—C231	−160.90 (8)
P3—Pt2—P4—C431	16.13 (8)	P1—Pt1—P2—C221	−97.89 (8)
Cl3—Pt2—P4—C431	−169.25 (8)	Cl2—Pt1—P2—C221	73.71 (8)
Cl4—Pt2—P4—C431	−126.32 (12)	P1—Pt1—P2—C217	145.58 (8)
P3—Pt2—P4—C417	136.63 (7)	Cl2—Pt1—P2—C217	−42.82 (8)
Cl3—Pt2—P4—C417	−48.74 (7)	C116—C111—C112—C113	1.7 (4)
Cl4—Pt2—P4—C417	−5.82 (14)	C117—C111—C112—C113	−176.5 (2)
C316—C311—C312—C313	1.2 (3)	C111—C112—C113—C114	−0.3 (4)
C317—C311—C312—C313	−179.8 (2)	C112—C113—C114—C115	−1.2 (5)
C311—C312—C313—C314	−0.9 (3)	C113—C114—C115—C116	1.4 (6)
C312—C313—C314—C315	0.0 (3)	C114—C115—C116—C111	0.0 (5)
C313—C314—C315—C316	0.6 (4)	C112—C111—C116—C115	−1.5 (4)
C312—C311—C316—C315	−0.6 (3)	C117—C111—C116—C115	176.7 (3)
C317—C311—C316—C315	−179.6 (2)	C112—C111—C117—P1	73.8 (3)
C314—C315—C316—C311	−0.3 (4)	C116—C111—C117—P1	−104.4 (2)
C316—C311—C317—P3	−126.29 (19)	C121—P1—C117—C111	59.62 (19)
C312—C311—C317—P3	54.8 (3)	C131—P1—C117—C111	170.05 (17)
C331—P3—C317—C311	173.28 (16)	Pt1—P1—C117—C111	−68.94 (18)
C321—P3—C317—C311	62.41 (18)	C131—P1—C121—C126	−0.6 (2)
Pt2—P3—C317—C311	−65.40 (17)	C117—P1—C121—C126	105.52 (19)
C331—P3—C321—C322	−1.0 (2)	Pt1—P1—C121—C126	−132.83 (16)
C317—P3—C321—C322	106.48 (18)	C131—P1—C121—C122	−174.73 (17)
Pt2—P3—C321—C322	−129.82 (16)	C117—P1—C121—C122	−68.63 (19)
C331—P3—C321—C326	−178.66 (16)	Pt1—P1—C121—C122	53.0 (2)
C317—P3—C321—C326	−71.16 (18)	C126—C121—C122—C123	−1.4 (3)
Pt2—P3—C321—C326	52.54 (18)	P1—C121—C122—C123	172.96 (18)
C326—C321—C322—C323	0.8 (3)	C121—C122—C123—C124	0.5 (4)
P3—C321—C322—C323	−176.83 (17)	C122—C123—C124—C125	0.7 (4)
C321—C322—C323—C324	−1.3 (3)	C123—C124—C125—C126	−0.9 (4)
C322—C323—C324—C325	0.8 (3)	C124—C125—C126—C121	0.0 (4)
C323—C324—C325—C326	0.1 (3)	C122—C121—C126—C125	1.2 (3)
C324—C325—C326—C321	−0.6 (3)	P1—C121—C126—C125	−172.96 (18)
C322—C321—C326—C325	0.1 (3)	C121—P1—C131—C132	−123.13 (18)
P3—C321—C326—C325	177.85 (17)	C117—P1—C131—C132	131.22 (18)
C321—P3—C331—C336	−125.49 (17)	Pt1—P1—C131—C132	12.69 (19)
C317—P3—C331—C336	127.65 (17)	C121—P1—C131—C136	60.0 (2)
Pt2—P3—C331—C336	6.60 (19)	C117—P1—C131—C136	−45.6 (2)
C321—P3—C331—C332	55.1 (2)	Pt1—P1—C131—C136	−164.15 (15)
C317—P3—C331—C332	−51.8 (2)	C136—C131—C132—C133	−0.7 (3)
Pt2—P3—C331—C332	−172.83 (16)	P1—C131—C132—C133	−177.66 (17)
C336—C331—C332—C333	1.7 (3)	C131—C132—C133—C134	0.7 (3)
P3—C331—C332—C333	−178.86 (18)	C132—C133—C134—C135	−0.4 (3)
C331—C332—C333—C334	−1.6 (4)	C133—C134—C135—C136	0.1 (4)
C332—C333—C334—C335	0.4 (4)	C134—C135—C136—C131	−0.1 (3)
C333—C334—C335—C336	0.6 (4)	C132—C131—C136—C135	0.4 (3)
C334—C335—C336—C331	−0.5 (3)	P1—C131—C136—C135	177.31 (17)
C332—C331—C336—C335	−0.7 (3)	C216—C211—C212—C213	0.5 (3)
P3—C331—C336—C335	179.86 (17)	C217—C211—C212—C213	−178.9 (2)
C416—C411—C412—C413	1.7 (3)	C211—C212—C213—C214	0.1 (4)
C417—C411—C412—C413	−179.9 (2)	C212—C213—C214—C215	−0.7 (4)
C411—C412—C413—C414	−1.0 (4)	C213—C214—C215—C216	0.7 (4)
C412—C413—C414—C415	−0.6 (5)	C214—C215—C216—C211	−0.1 (4)
C413—C414—C415—C416	1.4 (5)	C212—C211—C216—C215	−0.6 (3)
C412—C411—C416—C415	−0.9 (4)	C217—C211—C216—C215	178.9 (2)
C417—C411—C416—C415	−179.3 (3)	C212—C211—C217—P2	−101.4 (2)
C414—C415—C416—C411	−0.7 (5)	C216—C211—C217—P2	79.1 (2)
C416—C411—C417—P4	−116.9 (2)	C231—P2—C217—C211	60.85 (18)
C412—C411—C417—P4	64.8 (2)	C221—P2—C217—C211	173.06 (16)
C421—P4—C417—C411	175.69 (16)	Pt1—P2—C217—C211	−64.85 (17)
C431—P4—C417—C411	66.66 (17)	C231—P2—C221—C222	−120.35 (18)
Pt2—P4—C417—C411	−63.51 (17)	C217—P2—C221—C222	131.82 (18)
C431—P4—C421—C426	−109.51 (18)	Pt1—P2—C221—C222	9.1 (2)
C417—P4—C421—C426	142.88 (17)	C231—P2—C221—C226	61.3 (2)
Pt2—P4—C421—C426	22.83 (19)	C217—P2—C221—C226	−46.5 (2)
C431—P4—C421—C422	68.46 (19)	Pt1—P2—C221—C226	−169.15 (17)
C417—P4—C421—C422	−39.16 (19)	C226—C221—C222—C223	−1.9 (3)
Pt2—P4—C421—C422	−159.21 (15)	P2—C221—C222—C223	179.77 (18)
C426—C421—C422—C423	−0.2 (3)	C221—C222—C223—C224	1.0 (4)
P4—C421—C422—C423	−178.19 (17)	C222—C223—C224—C225	0.4 (4)
C421—C422—C423—C424	1.0 (3)	C223—C224—C225—C226	−0.8 (4)
C422—C423—C424—C425	−0.5 (3)	C224—C225—C226—C221	−0.1 (4)
C423—C424—C425—C426	−0.8 (3)	C222—C221—C226—C225	1.4 (3)
C424—C425—C426—C421	1.6 (3)	P2—C221—C226—C225	179.72 (18)
C422—C421—C426—C425	−1.1 (3)	C221—P2—C231—C236	−4.0 (2)
P4—C421—C426—C425	176.93 (17)	C217—P2—C231—C236	104.22 (19)
C421—P4—C431—C432	8.6 (2)	Pt1—P2—C231—C236	−131.67 (17)
C417—P4—C431—C432	116.31 (18)	C221—P2—C231—C232	175.15 (17)
Pt2—P4—C431—C432	−119.17 (16)	C217—P2—C231—C232	−76.59 (18)
C421—P4—C431—C436	−170.04 (17)	Pt1—P2—C231—C232	47.52 (19)
C417—P4—C431—C436	−62.28 (19)	C236—C231—C232—C233	1.0 (3)
Pt2—P4—C431—C436	62.23 (19)	P2—C231—C232—C233	−178.27 (17)
C436—C431—C432—C433	−0.5 (3)	C231—C232—C233—C234	−0.1 (3)
P4—C431—C432—C433	−179.09 (17)	C232—C233—C234—C235	−0.8 (3)
C431—C432—C433—C434	0.7 (3)	C233—C234—C235—C236	1.0 (4)
C432—C433—C434—C435	−0.3 (4)	C234—C235—C236—C231	−0.1 (4)
C433—C434—C435—C436	−0.5 (4)	C232—C231—C236—C235	−0.8 (3)
C434—C435—C436—C431	0.7 (4)	P2—C231—C236—C235	178.35 (18)
C432—C431—C436—C435	−0.2 (3)	Cl9B—Cl0A—C3—Cl9A	8.4 (7)
P4—C431—C436—C435	178.39 (17)	Cl9B—Cl0A—C3—Cl	48.7 (17)
P2—Pt1—P1—C121	22.16 (9)	Cl0A—Cl9B—C3—Cl9A	−22.6 (19)
Cl1—Pt1—P1—C121	−164.58 (9)	Cl0A—Cl9B—C3—Cl	−30.5 (13)
